# The role of illness‐related cognition in the relationships between resilience and depression/anxiety in nasopharyngeal cancer patients

**DOI:** 10.1002/cam4.6688

**Published:** 2023-11-22

**Authors:** Shenghao Wang, Yang Deng, Yuan Zhang, Vivian Yawei Guo, Bo Zhang, Xi Cheng, Meiqi Xin, Yuantao Hao, Fengsu Hou, Jinghua Li

**Affiliations:** ^1^ Department of Medical Statistics, School of Public Health Sun Yat‐sen University Guangzhou China; ^2^ Sun Yat‐sen Global Health Institute Sun Yat‐sen University Guangzhou China; ^3^ Department of Radiation Oncology Sun Yat‐sen University Cancer Center, State Key Laboratory of Oncology in South China, Collaborative Innovation Center for Cancer Medicine, Guangdong Key Laboratory of Nasopharyngeal Carcinoma Diagnosis and Therapy Guangzhou China; ^4^ Department of Rehabilitation Sciences The Hong Kong Polytechnic University Hong Kong China; ^5^ Peking University Center for Public Health and Epidemic Preparedness & Response Peking University Beijing China; ^6^ Key Laboratory of Epidemiology of Major Diseases (Peking University) Ministry of Education Beijing China; ^7^ Department of Public Health Shenzhen Kangning Hospital Shenzhen China

**Keywords:** anxiety, depression, illness perception, meaning in life, resilience, stigma

## Abstract

**Objective:**

Resilience has been reported as an important predictor of better mental health and prognoses in cancer patients, while its mechanisms were not clearly elucidated. In this study, we surveyed a large sample of nasopharyngeal carcinoma patients to investigate the mediating role of illness‐related cognition (illness perception, stigma and meaning in life) on the associations between resilience and symptoms of anxiety and depression.

**Methods:**

This cross‐sectional study involved 773 participants diagnosed with nasopharyngeal carcinoma. Participants completed a self‐reported structured questionnaire to assess their illness perception, stigma and meaning in life, resilience and symptoms of anxiety and depression. Structural equation models (SEM) were employed to explore the relationship between resilience and symptoms of anxiety and depression in the entire sample, as well as in two subgroups: Subgroup I (0–1 year since diagnosis), and Subgroup II (over 1 year since diagnosis).

**Results:**

In the entire sample, after adjusting for potential confounders, illness perception, stigma and meaning in life were found to mediate the protective effect of resilience on symptoms of depression (mediating effect proportion: 65.25%) and anxiety (mediating effect proportion: 67.63%). In Subgroup I, direct effects were dominant in the associations between resilience and symptoms of anxiety (mediating effect proportion: 37.95%) and depression (mediating effect proportion: 29.13%). However, in Subgroup II, the associations between resilience and symptoms of anxiety (mediating effect proportion: 98.92%) and depression (mediating effect proportion: 81.04%) were completely mediated.

**Conclusions:**

Our study suggests that direct and indirect effects of resilience on depression and anxiety dominate in early periods (0–1 year) and long‐term periods (over 1 year) following the cancer diagnosis, respectively. The findings indicate that comprehensive intervention considering both the direct effect of resilience in early stages (e.g., health education prescription and social support groups) and the indirect effects of illness cognition in long‐term periods (e.g., cognitive behavioral therapies) are likely to yield the most favorable outcomes for cancer patients.

## Graphical abstract

The study found that illness perception, stigma, and meaning in life mediated the protective effect of resilience on symptoms of depression and anxiety among nasopharyngeal carcinoma patients. Further, the direct and indirect effects dominate in early‐periods (0‐1year) and long‐term periods (over 1 year) since cancer diagnosis, respectively. The findings indicated that cognation‐related interventions based on the meaning dimension in resilience (e.g., cognitive behavioral therapies) played crucial roles in long‐term periods since cancer diagnosis, while interventions based on the plasticity and sociality dimensions in resilience (e.g., health education prescription and social support groups) should be considered in early‐term since cancer diagnosis.

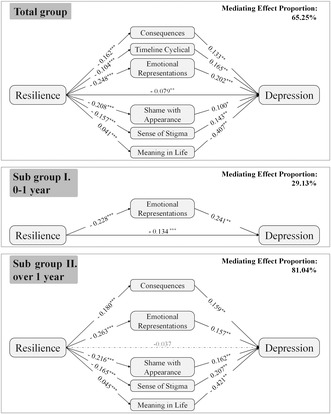

## 1 INTRODUCTION

Nasopharyngeal carcinoma (NPC) is an epithelial carcinoma that originating from the nasopharyngeal mucosal lining. Patients with NPC often face challenges related to appearance and function due to the disease and associated treatments; these challenges can contribute to increased stress, internalized stigma, depression, and anxiety.^1^, ^2^ Poor mental health not only impacts the quality of life for cancer patients but also affects their long‐term prognoses and overall survival. Previous studies have reported the relationships between depressive symptoms and shorter survival, higher rates of chemoradiation interruption, and poorer treatment response among cancer patients.^3^, ^4^, ^5^, ^6^

Resilience plays an important role in preventing depression and anxiety among individuals experiencing trauma. It is defined as the process that empowers individuals to quickly recover and regain their pre‐crisis status following exposure to trauma.^7^ A study conducted on head and neck cancer (HNC) patients in Pakistan found an inverse correlation between resilience, anxiety, and depression.^8^ Furthermore, a systematic review indicated that patients with somatic symptoms who exhibited better mental status, including lower levels of anxiety, depression, and pain, were associated with greater resilience.^9^ Affective responses provided valuable insights into comprehending the impact of resilience on mental health. Studies have indicated that affective well‐being and positive affect play significant mediating roles in the relationship between resilience and mental health in patients with breast cancer as well as gastric cancer.^10^, ^11^ Another study involving a heterogeneous group of cancer patients also reported the crucial mediating role of emotion regulation.^12^

Cognitive restructuring may be another factor that plays a critical role in the process of resilience improving mental health. According to Kumpfer's resilience framework, resilience can provide individuals with a protective environment to cope with stressful events by engaging in cognitive restructuring process during their interaction with the context.^13^ Feldman described resilience from an affiliative neuroscience perspective,^14^ emphasizing three progressive core features: plasticity, sociality, and meaning. Plasticity and sociality focus on the development of the nervous system and social networks; while meaning focuses on giving significance and inspiring strength in the face of human suffering, indicating its role in cognitive reconstruction.^14^ Studies have reported correlations between illness‐related cognitive processes (such as tending to pay attention to cancer‐related information and draw negative conclusions) and mental health with resilience in cancer patients.^15^ Illness perceptions and stigma have been reported as ones of most important illness‐related cognitive factors that impact mental health among NPC patients.^16^, ^17^, ^18^ Illness perceptions, which encompass patients' cognitive, emotional, and affective responses to their symptoms and disease, are associated with mental health outcomes in individuals with cancer.^19^ Stigma refers to the sense of being rejected, blamed, or devalued due to possessing an attribute that society labels as undesirable.^20^ The diagnosis of nasopharyngeal carcinoma along with its late toxicities (such as trismus, xerostomia, and brain injury) contributes to feelings of stigma among patients,^21^ negatively affecting their rehabilitation, quality of life, and mental health.^16^, ^17^

Moreover, disease‐related cognitive processes among patients and their impact on mental health may undergo changes over time following a cancer diagnosis. According to the Corbin and Strauss Chronic Illness Trajectory model, patients enter the coping phase and may go through stages of shock, defense mechanisms, and anger following their diagnosis.^22^ In a longitudinal study, illness perceptions are reported to account for varying proportions of the variance in anxiety and depressive symptoms at 3 and 12 months of follow‐up among cancer patients.^23^ Patients' affective well‐being, such as meaning in life,^24^ may require several months or even years to undergo positive transformations. This implies that the associations between illness perceptions, stigma, meaning in life, resilience and mental health may vary across different periods following a cancer diagnosis. Resilience has served as a vital foundation for the development of cognitive behavioral therapies and mindfulness‐based interventions in clinical practice.^25^, ^26^ However, there is a lack of evidence that releases the mechanisms and pathways through which resilience improves mental health in different periods following cancer diagnosis in NPC patients, despite their substantial clinical and public health implications.

In this study, we aimed to investigate the mediating effects of illness perception, stigma and meaning in life on the relationship between resilience and symptoms of anxiety and depression among patients with nasopharyngeal carcinoma. Furthermore, we sought to examine whether this association varies between the early periods and long‐term periods following cancer diagnosis and treatment.

## 2 METHODS

### 2.1 Sample and procedure

We conducted a cross‐sectional survey at the Sun Yat‐sen University Cancer Center (Guangzhou, China) between January 2020 and January 2023. Convenience sampling was employed to recruit a total of 831 patients diagnosed with nasopharyngeal carcinoma (NPC). The inclusion criteria were (1) having a confirmed NPC diagnosis by pathological biopsy; (2) being aged 18 and over; and (3) being able to read and communicate in Mandarin. Participants with severe mental disorders (e.g., schizophrenia or intellectual disability), other malignancies, or severe physical conditions (such as stoke) were excluded.

The participants were informed that declining to complete the survey would not impact their access to medical services. Written informed consents have been obtained from all participants prior to the survey. The study received approval from the Ethics Committee of Sun Yat‐sen University (No. 2019‐145).

### 2.2 Measurements

#### 2.2.1 Sociodemographic and clinical characteristics

Sociodemographic information (including age, gender, and monthly household income), behavioral information (ever smoking, and ever drinking), and clinical characteristics (stage of diagnosis and date of diagnosis) were collected. Furthermore, the time interval from diagnosis to survey was defined as the interval between the date of investigation and the date of diagnosis.

#### 2.2.2 Depression symptoms

The Chinese version of the Patient Health Questionnaire–9 (PHQ‐9)^27^ was utilized to assess participants' depressive symptoms, and it is a validated self‐report scale with good reliability and validity.^28^ The PHQ‐9 consists of nine items and measures the frequency of depression symptoms experienced over the past 2 weeks using a 4‐point Likert scale ranging from “not at all” to “almost every day”. The total score ranges from 0 to 27, with the cut‐off values of 5, 10, and 15 indicating mild, moderate, and severe depressive symptoms, respectively. The Cronbach's *α* was 0.917 in this study.

#### 2.2.3 Anxiety

The Chinese version of the General Anxiety Disorder scale (GAD‐7) was utilized to assess participants' anxiety symptoms, and it is a validated self‐report scale with good reliability and validity.^29^, ^30^ The GAD‐7 consists of seven items and measures the frequency of anxiety symptoms in the past 2 weeks using a 4‐point Likert scale ranging from “not at all” to “almost every day”. The total score ranges from 0 to 21, with the cut‐off values of 5, 10, and 15 indicating mild, moderate, and severe anxiety symptoms, respectively. The Cronbach's *α* was 0.949 in this study.

#### 2.2.4 Resilience

The Chinese version of the 10‐item Connor–Davidson Resilience Scale (CD‐RISC10) was utilized to assess participants' resilience, and it is a validated self‐report scale with good reliability and validity.^31^, ^32^ The CD‐RISC10 is a self‐report instrument in which items are scored on a 5‐point Likert scale ranging from 0 (“not true at all”) to 4 (“true nearly all the time”). The total score ranges from 0 to 40, with higher scores indicating a greater level of resilience. The Cronbach's *α* was 0.962 in this study.

#### 2.2.5 Illness perception

The 38‐item Illness Perception Questionnaire Revision (IPQ‐R) was utilized to assess participants' illness perceptions, and the Chinese version has been validated in Chinese populations with good reliability and validity.^33^, ^34^ Participants were instructed to rate the items on a 5‐point Likert scale from 1 (“strongly disagree”) to 5 (“strongly agree”). The scale consists of seven dimensions that evaluate perceptions and counter‐perceptions related to coping with cancer, including: timeline chronic–acute (perceived degree of chronicity of the disease), consequences (consequences of the disease for different areas of the patient's life), personal control (perceived ability to control the disease), treatment control (perception of the extent to which treatment can control the disease), illness coherence (general understanding of the disease), timeline periodicity (perceived course of the disease from the periodic appearance of symptoms), and emotional representations (emotional burden caused by the disease). The total scores ranges from 38 to 190, and higher scores indicating higher levels of the relevant dimension. For example, high scores on the emotional representation items indicated high levels of emotional stress. The Cronbach's *α* was 0.729 in this study.

#### 2.2.6 Meaning in life

Participants were asked to respond to the statement “My life has a clear sense of purpose?” using a 7‐point Likert scale, ranging from 1 (“absolutely untrue”) to 7 (“absolutely true”). A higher score indicates a greater sense of meaning in life. The question was adapted from the Meaning in Life Questionnaire (MLQ),^35^ and its Chinese version has been validated with good reliability and validity in Chinese populations.^36^

#### 2.2.7 Shame and stigma

The Shame and Stigma Scale (SSS) was utilized to assess participants' perception of shame and stigma, and its Chinese version has been validated in Chinese HNC populations with good reliability and validity.^37^, ^38^, ^39^ The 20‐item self‐report scale adopts a 5‐point Likert scale, ranging from 0 (“never”) to 4 (“always”). The scale consists of four dimensions: shame with appearance, sense of stigma, regret, social/speech concerns. Higher scores indicated more shame and stigma. The Cronbach's *α* was 0.903 in this study.

### 2.3 Statistical analysis

The descriptive statistics are presented in counts, percentage, mean, and standard deviation (SD).

Structural equation models (SEM) were conducted in two steps. First, we constructed main models using the full sample set (**Total group**). Based on previous studies, age, gender, and monthly household income, ever smoking, ever drinking, stage of diagnosis, time interval from diagnosis to survey were controlled as potential confounders. Subsequently, we performed a stratified analysis based on the time interval from diagnosis to survey. Two subsets were derived from the full sample set (**Subgroup I: 0–1 year and Subgroup II: over 1 year**). The main models were refitted in Subgroup I and Subgroup II to assess whether there were any differences in the mediating effects between in the early periods and long‐term periods since cancer diagnosis.

A bootstrap approach (5000 times) was used to estimate the parameters and their 95% confidence intervals in our study. Model fit was examined using the comparative fit index (CFI), goodness of fit index (GFI), and root mean square error of approximation (RMSEA). CFI and GFI values from above 0.90 to above 0.95 and RMSEA values less than 0.08 to less than 0.05 are indicative of acceptable to good fit.

All statistical analyses were developed in R (version 4.1.3) with the package “lavaan” for the mediation.

## 3 RESULTS

### 3.1 Descriptive statistics and correlations

There were 773 valid questionnaires in the present study, with a response rate of 93%. Among the participants, 535 (69.2%) were male, while 238 (30.8%) were female. The age of the participants ranged from 18 to 85, with a mean of 47.2 (SD = 11.5). The mean time since cancer diagnosis was 2.68 (SD = 1.80) years, and the majority of the participants (59.8%) had been diagnosed for more than 1 year. There were 342 participants (44.2%) reported mild to severe symptoms of depression, with a mean score of 5.15 (SD = 5.49) on the PHQ‐9. And there were 273 participants (35.3%) reported mild to severe anxiety symptoms, with a mean score of 3.65 (SD = 4.44) on the GAD‐7. When comparing the different time intervals since cancer diagnosis, a lower proportion of patients in the long‐term periods reported symptoms of depression (41.8%) and anxiety (32.9%) compared to those in the early periods (depression: 47.9%, anxiety: 38.9%). (Table 1) Additionally, significant correlations were observed among almost all the variables (Figure S1).

**TABLE 1:** Demographic, behavior, clinical, and psychosocial characteristics (*N* = 773).

	Time since diagnosis		Total
	0–1 year (*n* = 311)	over 1 year (*n* = 462)	
Demographic			
Age			
≤30	27 (8.7)	39 (8.4)	66 (8.5)
31–40	66 (21.2)	99 (21.4)	165 (21.3)
41–50	93 (29.9)	148 (32.0)	241 (31.2)
51–60	84 (27.0)	120 (26.0)	204 (26.4)
≥61	41 (13.2)	56 (12.1)	97 (12.5)
Mean (SD)	47.50 (11.79)	46.92 (11.37)	47.16 (11.54)
Gender			
Female	87 (28.0)	151 (32.7)	238 (30.8)
Male	224 (72.0)	311 (67.3)	535 (69.2)
Family income (CNY/Month)			
≤3000	89 (28.6)	120 (26.0)	209 (27.0)
3001–5000	82 (26.4)	157 (34.0)	239 (30.9)
5001–10,000	83 (26.7)	111 (24.0)	194 (25.1)
≥10,001	57 (18.3)	74 (16.0)	131 (16.9)
Behavior			
Ever drinking			
No	277 (89.1)	434 (93.9)	711 (92.0)
Yes	34 (10.9)	28 (6.1)	62 (8.0)
Ever smoking			
No	195 (62.7)	316 (68.4)	511 (66.1)
Yes	116 (37.3)	146 (31.6)	262 (33.9)
Clinical			
Stage of diagnosis			
Earlier (I–II)	34 (10.9)	43 (9.3)	77 (10.0)
Advanced (III–IV)	277 (89.1)	419 (90.7)	696 (90.0)
Months since diagnosis (mean (SD))	5.46 (3.11)	34.03 (20.89)	22.53 (21.47)
Psychosocial			
Resilience (mean (SD))	24.95 (8.95)	25.27 (8.36)	25.14 (8.60)
Illness Perception (mean (SD))			
Timeline acute/chronic	15.10 (4.56)	15.97 (4.63)	15.62 (4.62)
Consequences	17.90 (4.66)	17.70 (4.53)	17.78 (4.58)
Personal control	19.86 (3.70)	20.39 (3.48)	20.18 (3.58)
Treatment control	18.27 (2.93)	18.09 (2.99)	18.16 (2.97)
Illness coherence	15.64 (3.79)	15.86 (3.79)	15.77 (3.79)
Timeline cyclical	9.97 (3.33)	10.02 (3.13)	10.00 (3.21)
Emotional representations.	17.08 (4.86)	17.09 (5.06)	17.09 (4.98)
Shame and Stigma (mean (SD))			
Shame with appearance	9.64 (5.87)	9.19 (5.50)	9.37 (5.65)
Sense of stigma	5.52 (5.44)	4.98 (5.02)	5.20 (5.20)
Regret	3.87 (3.24)	3.57 (2.96)	3.69 (3.08)
Speech/social concerns	3.14 (2.47)	3.08 (2.42)	3.11 (2.44)
Meaning in life (mean (SD))	6.03 (1.29)	6.07 (1.32)	6.06 (1.31)
Depressive symptoms			
No	162 (52.1)	269 (58.2)	431 (55.8)
Mild	94 (30.2)	121 (26.2)	215 (27.8)
Moderate to severe	55 (17.7)	72 (15.6)	127 (16.4)
Mean (SD)	5.49 (5.59)	4.92 (5.41)	5.15 (5.49)
Anxiety			
No	190 (61.1)	310 (67.1)	500 (64.7)
Mild	87 (28.0)	114 (24.7)	201 (26.0)
Moderate to severe	34 (10.9)	38 (8.2)	72 (9.3)
Mean (SD)	3.93 (4.62)	3.45 (4.31)	3.65 (4.44)

### 3.2 Structural equation models using the full sample set (Total group)

**FIGURE 1:**
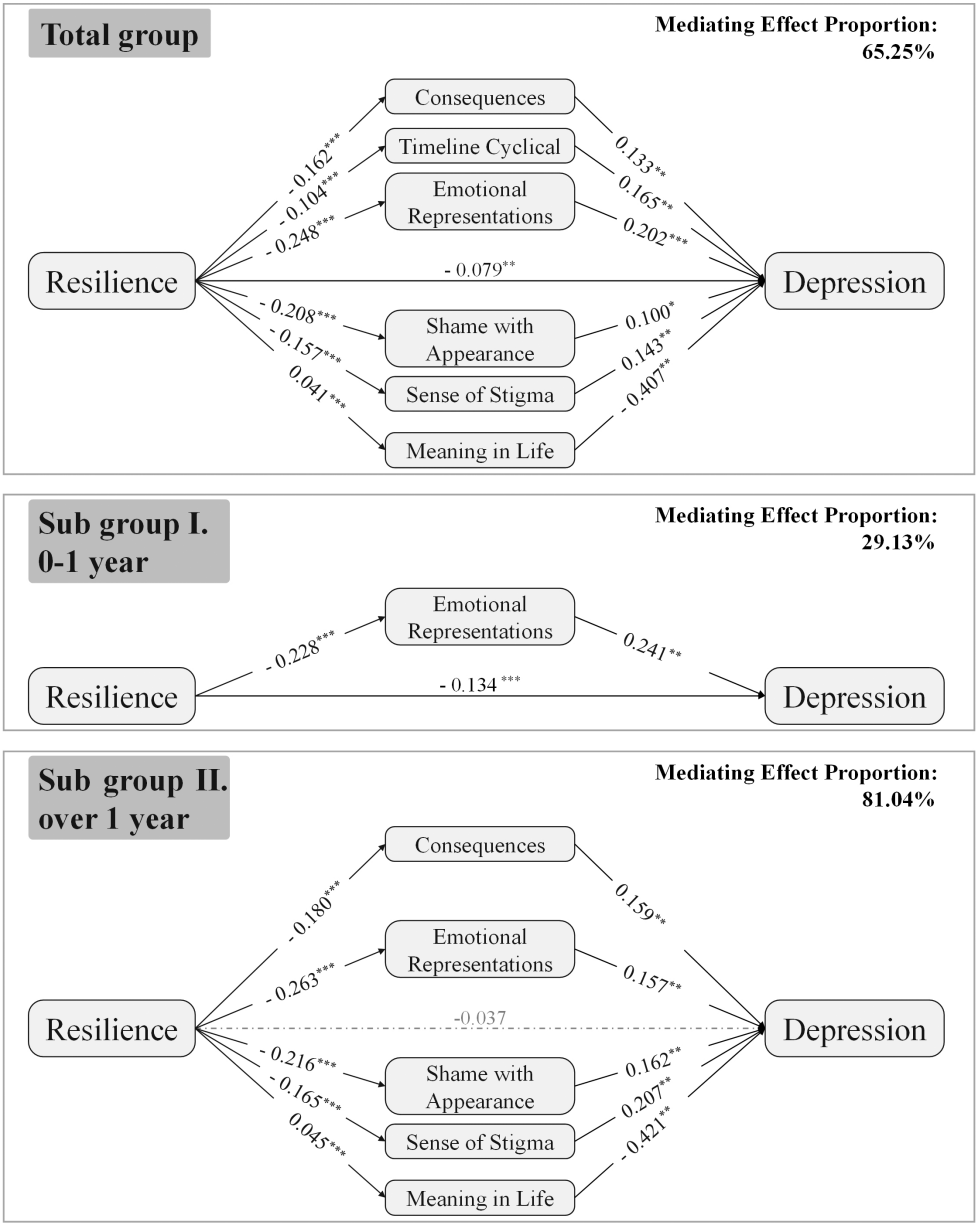
Structural model and path coefficients of the mediating effects of illness perception, stigma, meaning in life on the relationship between resilience and symptoms of depression. *p < 0.05, **p < 0.01, ***p < 0.001.

The SEM model constructed with symptoms of depression as the outcome variable provided an acceptable fit to the full sample set (χ^2^ = 320.6, df = 151, CFI = 0.957, GFI = 0.957, RMSEA = 0.038). (Table S1) Illness perception, stigma and meaning in life mediated the effect of resilience on symptoms of depression (mediating effect proportion 65.25%) after controlling for potential confounders. Resilience was observed to improve symptoms of depression by improving illness perception (consequences, timeline cyclical, and emotional representations), reducing stigma (shame with appearance and sense of stigma), and promoting meaning in life. (Tables 2 and S2, Figure 1).

**TABLE 2:** Direct, indirect, and total effects of resilience on symptoms of depression and anxiety.

	Bate	95% CI	*p*
Resilience → Depression			
Total group			
Direct effect	−0.079	(−0.129, −0.033)	0.001
Indirect effect	−0.149	(−0.185, −0.116)	<0.001
Total effect	−0.228	(−0.283, −0.178)	<0.001
Proportion			65.25%
Subgroup I. 0–1 year			
Direct effect	−0.134	(−0.220, −0.058)	0.001
Indirect effect	−0.055	(−0.096, −0.019)	0.005
Total effect	−0.189	(−0.273, −0.113)	<0.001
Proportion			29.13%
Subgroup II. over 1 year			
Direct effect	−0.037	(−0.095, 0.021)	0.206
Indirect effect	−0.158	(−0.207, −0.112)	<0.001
Total effect	−0.195	(−0.259, −0.133)	<0.001
Proportion			81.04%
Resilience → Anxiety			
Total group			
Direct effect	−0.042	(−0.084, −0.001)	0.047
Indirect effect	−0.087	(−0.115, −0.062)	<0.001
Total effect	−0.129	(−0.175, −0.087)	<0.001
Proportion			67.63%
Subgroup I. 0–1 year			
Direct effect	−0.087	(−0.157, −0.022)	0.012
Indirect effect	−0.053	(−0.088, −0.021)	0.002
Total effect	−0.140	(−0.212, −0.074)	<0.001
Proportion			37.95%
Subgroup II. over 1 year			
Direct effect	−0.001	(−0.053, 0.048)	0.960
Indirect effect	−0.119	(−0.159, 0.081)	<0.001
Total effect	−0.120	(−0.174, −0.067)	<0.001
Proportion			98.92%

**FIGURE 2:**
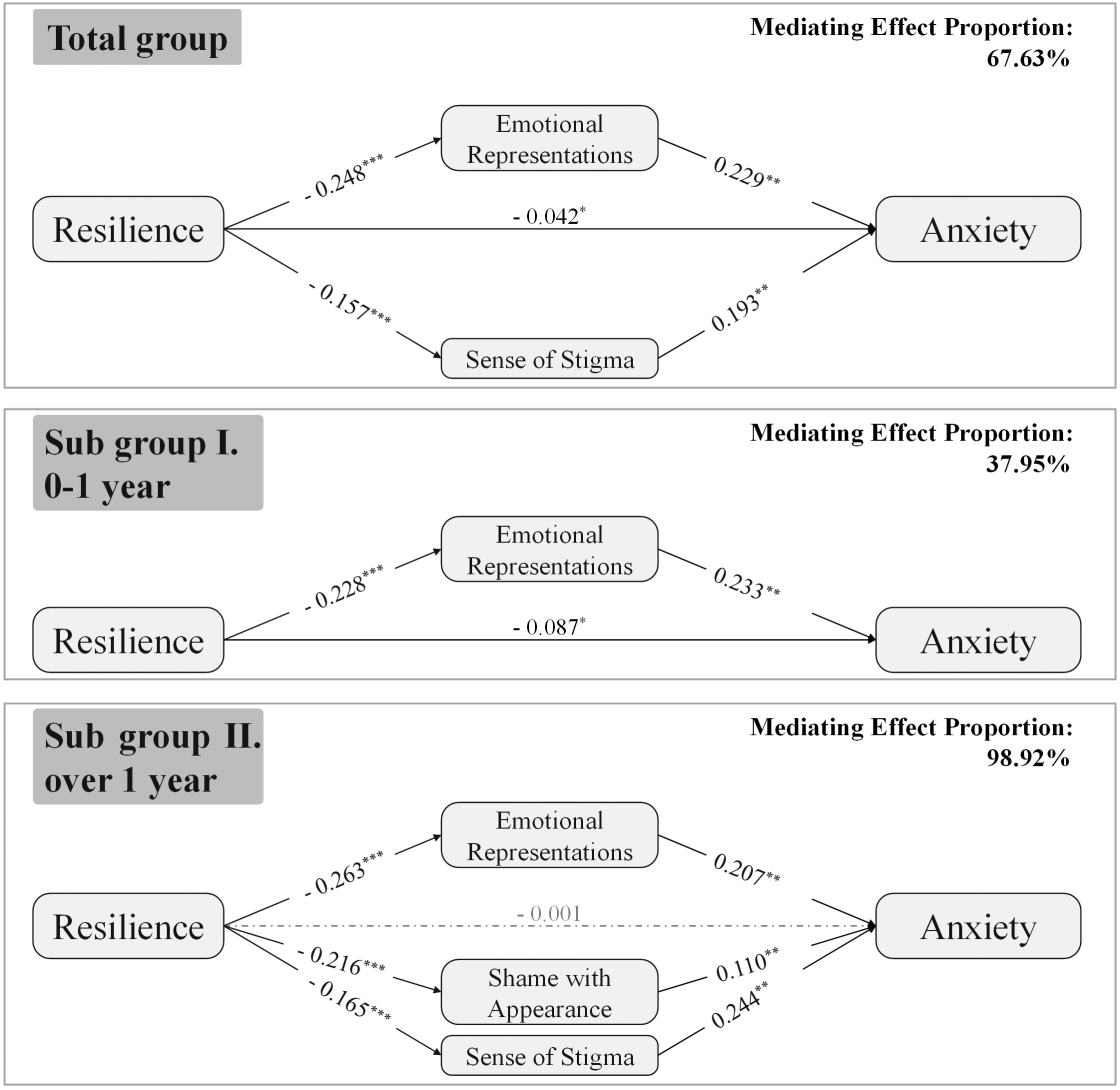
Structural model and path coefficients of the mediating effects of illness perception, stigma on the relationship between resilience and symptoms of anxiety. *p < 0.05, **p < 0.01, ***p < 0.001.

Similarly, the SEM model constructed with anxiety as the dependent variable provided an acceptable fit to the full sample set (χ^2^ = 318.4, df = 151, CFI = 0.957, GFI = 0.957, RMSEA = 0.038). (Table S1) The relationship between resilience and anxiety was mediated (mediating effect proportion 67.63%) by illness perception (emotional representations) and stigma (sense of stigma). In contrast, meaning in life was not observed as a mediator in the relationship between resilience and anxiety. (Tables 2 and S2, Figure 2).

### 3.3 Stratified analysis in two subgroups (Subgroup I: 0–1 year and Subgroup II: over 1 year)

We observed that main models refitted in Subgroup I and Subgroup II performed a good fit (χ^2^ = 217.1 ~ 227.9, df = 151, CFI = 0.954–0.970, GFI = 0.930–0.949, RMSEA = 0.033–0.039). (Table S1).

Among patients in early periods since cancer diagnosis (Subgroup I), we found the direct effects were dominate and only illness perception (emotional representations) mediated the effect of resilience on symptoms of depression (mediating effect proportion 29.13%) and anxiety (mediating effect proportion 37.95%). (Tables 2 and S2, Figures 1 and 2).

In contrast, the dominance of indirect effects was observed among patients in long‐term periods since cancer diagnosis (Subgroup II). The association between resilience and symptoms of depression was completely mediated (mediating effect proportion 81.04%) by illness perception, stigma, and meaning in life. We found that the stigma and meaning in life only mediate the relationship between resilience and symptoms of depression in Subgroup II. Similarly, the association between resilience and anxiety was completely mediated (mediating effect proportion 98.92%) by illness perception, and stigma. And the stigma was identified as a mediator in the effect of resilience on anxiety only in Subgroup II. (Tables 2 and S2, Figures 1 and 2).

## 4 DISCUSSION

We found a higher proportion of depression and anxiety symptoms among participants in early periods (0–1 year) since their cancer diagnosis. Furthermore, our findings revealed that illness perception, stigma, and meaning in life mediated the protective effect of resilience on symptoms of depression and anxiety among NPC patients. Importantly, the time interval since diagnosis was found to have a significant influence on the mediating process, with direct and indirect effects being more prominent early periods (0–1 year) and long‐term periods (over 1 year) since cancer diagnosis, respectively. These findings highlight the significance of implementing cognitive interventions, such as cognitive behavior therapy, and suggest that the need for additional support interventions aimed at improving the mental health of patients in the early period following their cancer diagnosis.

In our survey, nearly half of the participants (47.9%) in early periods (0–1 year) since cancer diagnosis reported mild to severe symptoms of depression. This proportion decreased to 41.8% among patients in long‐term periods. Current literature reported that there are 22%–57% of HNC patients suffering depression.^40^ The decreasing trend in our study aligns with findings from a longitudinal study conducted in HNC patients, where depression levels were higher in the early post‐diagnosis period (42% in month 1) due to factors such as treatment and body image concerns, and then gradually decreased over time (29% in month 6).^41^ Similarly, we found a decreasing trend in the proportion of anxiety symptoms in both subgroups. In the early periods, 38.9% of patients reported mild to severe anxiety, which decreased to 32.9% in the long‐term periods. A prospective longitudinal study conducted in HNC patients also implied a similar trend, with the prevalence of clinical anxiety symptoms reported as 32.0% and 12.6% at baseline and 12 months.^42^ These findings highlight the dynamic nature of psychological distress in patients with HNC, with higher levels of depression and anxiety observed in the early post‐diagnosis period and a gradual decrease over time.

We found illness perception, stigma and meaning in life mediated the negative relations between resilience and symptoms of depression. As hypothesized, resilience was associated with lower levels of stigma and negative illness‐related perception, while promoting positive illness‐related perceptions and the process of meaning making. These factors collectively contributed to reducing the level of depression. Specifically, the dimensions of consequences and emotional representations within illness perception were found to be important in influencing the mental health of cancer patients, consistent with previous studies that have reported their association with anxiety and depression.^18^, ^43^ Further, we also found two dimensions of stigma (shame with appearance and sense of stigma) were significant mediators. A study conducted on HNC patients also reported that satisfaction with one's appearance was an important predictor of depressive symptoms.^41^ Given that patients with HNC often experience disfigurements associated with treatment, such as changes in appearances, it is understandable that decreased satisfaction with appearance and interference with self‐esteem can contribute to depressive symptoms.^44^ In this study, meaning in life was another important mediating factor between resilience and depression. It was consistent with a review that suggested a negative association between meaning in life and distress, including anxiety and depressive symptoms, in cancer patients.^45^ Stressful life events, such as cancer diagnosis, could initiate the meaning‐making process, and successful meaning‐making may lead to reduced distress.^46^ Regarding anxiety, we found that illness perception and stigma mediated the relationship between resilience and anxiety. However, meaning in life did not mediate this relationship. This finding is consistent with a previous multinational study that reported a nonsignificant correlation between meaning in life and anxiety.^47^ In the context of cancer patients, benefit finding is related to more intrusive and avoidant thoughts about the illness,^48^ which may explain the inconsistent findings regarding the relationship between the meaning in life and anxiety. These findings provide insight into the mechanisms through which resilience influences anxiety and depression in patients with nasopharyngeal carcinoma (NPC). The results support the implementation of cognitive therapy and mindfulness‐based interventions in clinical practice to enhance resilience and improve mental health outcomes for NPC patients.

Further, we conducted a stratified analysis based on the time interval from diagnosis to survey, examining the associations between resilience, mental health outcomes, and cognitive processes of illness in patients within the early and long‐term periods, as well as the entire sample. Consistent with our findings, a cohort study also reported that resilience was associated with anxiety, depression, and cognitive processes of illness at both baseline and 6‐month follow‐up.^15^ Specifically, in the early periods (0–1 year) after diagnosis, resilience had a direct effect on symptoms of depression and anxiety. However, in the long‐term periods (over 1 year) following cancer diagnosis, the relationships between resilience and symptoms of depression and anxiety were completely mediated by illness perception, stigma, and meaning in life. One possible explanation for the higher levels of depressive symptoms in the early periods after diagnosis is the close relationship between somatic symptoms and the inflammatory response associated with the disease and its treatment.^49^ There are shared inflammatory mechanisms implicated in both depression and cancer such as the increasing IL‐6 and TNF‐α levels, furthermore, treatments for cancer can promote cancer‐related pain and further inflammation by enhancing cytokine production by noncancerous cells.^50^, ^51^ A trajectory study of depressive symptoms and immunity in cancer survivors reported a co‐occurrence of changes (recovery) in psychological and innate immunity markers from diagnosis to 18 months.^52^ And after about 8 months of declining depressive symptom declines in all patients, the trend in depression levels displayed significant individual differences, with some individuals experiencing an increase in depressive symptoms while others continued to decrease.^52^ Another explanation may be that cognitive processes and meaning‐finding take time to occur in patients after the cancer diagnosis.^22^, ^53^ Several studies have reported variations in illness perception at different times after cancer diagnosis, resulting in different proportions of the variance in anxiety and depressive symptoms.^23^, ^54^ Similarly, stigma has been reported to vary across periods following cancer diagnosis and treatment, closely relating to mental health outcomes.^17^, ^55^ These findings provide information to a deeper understanding of the psychological processes and inform clinical practice for cancer patients following their diagnosis.

### 4.1 Clinical implications

Resilience has received significant attention in clinical practice as an important positive psychology factor. Various cognitive behavioral therapies and mindfulness‐based interventions (e.g., mindfulness awareness practices and internet‐based mindfulness‐based cognitive therapy), which are designed based on resilience theories, have shown promising results in improving mental health and overall survival outcomes in cancer patients.^25^, ^26^ Our study adds evidence supporting these interventions, as we found that resilience improves mental health in cancer patients by positively influencing long‐term cognitive processes related to the disease and meaning‐making. Further, we found resilience mainly had a direct effect on reducing symptoms of depression and anxiety in the early periods (0–1 year) following a cancer diagnosis, and patients could not benefit from the illness‐related cognition improvement after diagnosis immediately. Unfortunately, patients' symptoms of depression and anxiety were more severe in early periods (0–1 year) after diagnosis. Therefore, it is essential to provide additional support (e.g., coping skills and social support) to improve resilience and mental health rapidly and reduce the potential risk of treatment failure and shorter survival in the short term following the patient's diagnosis.^5^ These interventions may involve implementing interventions such as health education prescription (an individualized non‐medical intervention to guide patients in their daily health management and improve their quality of life)^56^ and encouraging participation in social support groups.^57^ Overall, adopting an integrated approach that addresses mental problems both in the early and long‐term periods of the disease, such as combining education prescription^56^ with cognitive behavioral therapies,^25^, ^26^ is likely to yield the most beneficial outcomes for patients.

### 4.2 Study limitations

The present study involved a large sample and added new understandings about resilience and mental health. However, our interpretation of the results was still limited by the following concerns. First, we were unable to estimate the causal relationship due to the cross‐sectional design. The recall and reporting biases in the self‐report questionnaires may have affected the results. Prospective longitudinal studies were required to further demonstrate causal effects. Second, the impact of COVID‐19 was not measured, with all participants recruited during the COVID‐19 pandemic period. Finally, the participants were only recruited from one cancer hospital in Guangzhou, China. However, the Sun Yat‐sen University Cancer Center admits patients from all over the country and Guangzhou is a large international city, which increase the generalizability of our results.

## 5 CONCLUSION

We found that illness perception, stigma, and meaning in life mediated the protective effect of resilience on symptoms of depression and anxiety in NPC patients. Further, we observed that direct and indirect effects dominate in early periods (0–1 year) and long‐term periods (over 1 year) following cancer diagnosis, respectively. These findings suggested that comprehensive interventions that encompass both early stages (e.g., health education prescription and social support groups) and long‐term periods (e.g., cognitive behavioral therapies) are likely to yield the most favorable outcomes for cancer patients.

## AUTHOR CONTRIBUTIONS

**Shenghao Wang:** Conceptualization (equal); data curation (equal); formal analysis (equal); writing – original draft (equal). **Yang Deng:** Conceptualization (equal); data curation (equal); visualization (equal). **Yuan Zhang:** Conceptualization (equal); data curation (equal); visualization (equal); writing – original draft (equal). **Vivian Yawei Guo:** Conceptualization (equal); data curation (equal); supervision (equal). **Bo Zhang:** Investigation (equal); supervision (equal). **Xi Cheng:** Investigation (equal); supervision (equal). **Meiqi Xin:** Supervision (equal); validation (equal); visualization (equal); writing – review and editing (equal). **Yuantao Hao:** Conceptualization (equal); writing – review and editing (equal). **Fengsu Hou:** Supervision (equal); validation (equal); writing – review and editing (equal). **Jinghua Li:** Conceptualization (equal); data curation (equal); formal analysis (equal); methodology (equal); writing – review and editing (equal).

## CONFLICT OF INTEREST STATEMENT

The authors declare that there is no conflict of interest with the conduct of this study.

## ETHICS STATEMENT

The study was started after obtaining permission from the Ethics Committee of Sun Yat‐sen University (No. 2019‐145).

## COMPLIANCE WITH ETHICAL STANDARDS

All procedures performed in studies involving the participants were in accordance with the ethical standards of the institutional and/or national research committee and with the 1964 Helsinki declaration and sent was obtained from all individual participants included in the study.

## CONSENT TO PARTICIPATE

Informed consent was obtained from all individual participants included in the study.

## Supporting information


Data S1.

## Data Availability

The data sets used during the current study are available from the corresponding author upon reasonable request.
