# Supplementary material for: The role of illness‐related cognition in the relationships between resilience and depression/anxiety in nasopharyngeal cancer patients
Source: Cancer Med. 2023 Nov 22;12(23):21408–18. doi: 10.1002/cam4.6688 (PMC10726906; doi:10.1002/cam4.6688)
Supplement: Supplementary file 1 — Data S1. [file CAM4-12-21408-s001.docx]

**Supplementary Materials**

1. **Pairwise correlation coefficients**

| 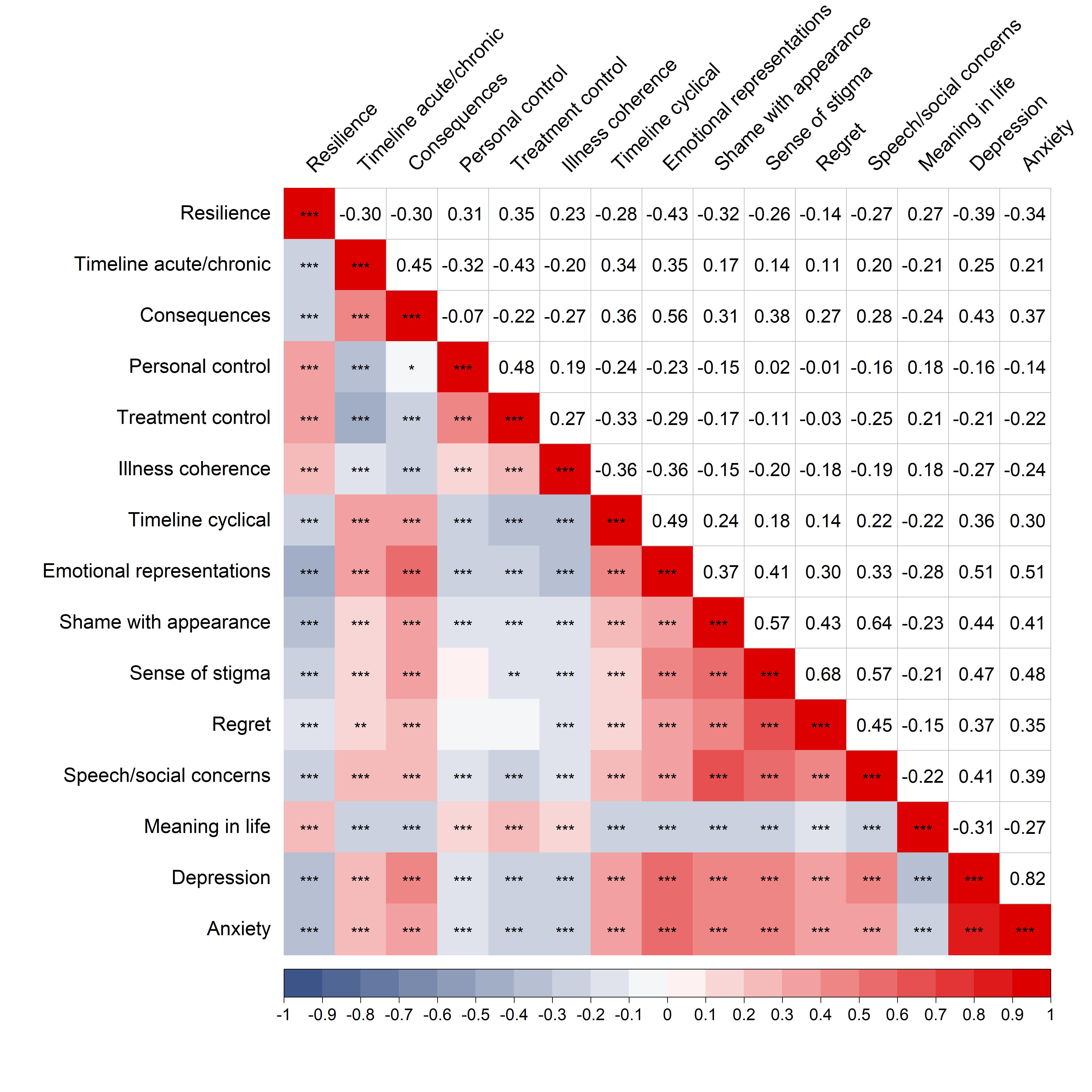 |
| --- |
| **p* < 0.05, ***p* <0.01, ****p* <0.001. |
| Fig S1. The heat map of pairwise correlation coefficients between the psychosocial variables |

**2. Structural equation model fit**

Table S1 The main fit evaluation index of structural equation model

|  | ***Chisq*** | ***df*** | ***Chisq/df*** | ***GFI*** | ***CFI*** | ***RMSEA*** |
| --- | --- | --- | --- | --- | --- | --- |
| **Depression** |  |  |  |  |  |  |
| *Total group* | 320.600 | 151 | 2.123 | 0.957 | 0.957 | 0.038 |
| *Subgroup I. 0-1 year* | 220.674 | 151 | 1.461 | 0.930 | 0.954 | 0.039 |
| *Subgroup II. over 1 year* | 225.839 | 151 | 1.496 | 0.949 | 0.970 | 0.033 |
| **Anxiety** |  |  |  |  |  |  |
| *Total group* | 318.413 | 151 | 2.109 | 0.957 | 0.957 | 0.038 |
| *Subgroup I. 0-1 year* | 217.079 | 151 | 1.438 | 0.931 | 0.955 | 0.038 |
| *Subgroup II. over 1 year* | 227.944 | 151 | 1.510 | 0.949 | 0.969 | 0.033 |

**3. The detail of structural equation model in the present study**

Table S2 The detail of direct, indirect, and total effects of resilience on depression and anxiety

|  | **Bate** | **95% *CI*** | ***P*** |
| --- | --- | --- | --- |
| **Resilience → Depression** |  |  |  |
| ***Total group*** |  |  |  |
| *Direct effect* | -0.079 | (-0.129, -0.033) | 0.001 |
| *Indirect effect 1* | 0.001 | (-0.013, 0.015) | 0.888 |
| *Indirect effect 2* | -0.022 | (-0.037, -0.007) | 0.004 |
| *Indirect effect 3* | -0.004 | (-0.017, 0.009) | 0.590 |
| *Indirect effect 4* | 0.008 | (-0.009, 0.024) | 0.370 |
| *Indirect effect 5* | -0.004 | (-0.014, 0.005) | 0.356 |
| *Indirect effect 6* | -0.017 | (-0.031, -0.004) | 0.009 |
| *Indirect effect 7* | -0.050 | (-0.075, -0.026) | <0.001 |
| *Indirect effect 8* | -0.021 | (-0.040, -0.004) | 0.026 |
| *Indirect effect 9* | -0.022 | (-0.042, -0.005) | 0.015 |
| *Indirect effect 10* | -0.006 | (-0.016, 0.002) | 0.183 |
| *Indirect effect 11* | -0.013 | (-0.029, 0.002) | 0.095 |
| *Indirect effect 12* | -0.017 | (-0.031, -0.005) | 0.009 |
| *Total effect* | -0.228 | (-0.283, -0.178) | <0.001 |
| ***Subgroup I. 0-1 year*** |  |  |  |
| *Direct effect* | -0.134 | (-0.220, -0.058) | 0.001 |
| *Indirect effect 1* | -0.001 | (-0.023, 0.022) | 0.909 |
| *Indirect effect 2* | -0.013 | (-0.033, 0.005) | 0.177 |
| *Indirect effect 3* | 0.009 | (-0.008, 0.028) | 0.334 |
| *Indirect effect 4* | 0.013 | (-0.010, 0.037) | 0.277 |
| *Indirect effect 5* | -0.007 | (-0.024, 0.008) | 0.370 |
| *Indirect effect 6* | -0.020 | (-0.045, 0.004) | 0.120 |
| *Indirect effect 7* | -0.055 | (-0.094, -0.019) | 0.004 |
| *Indirect effect 8* | -0.007 | (-0.034, 0.017) | 0.564 |
| *Indirect effect 9* | -0.009 | (-0.039, 0.018) | 0.538 |
| *Indirect effect 10* | -0.004 | (-0.019, 0.007) | 0.562 |
| *Indirect effect 11* | -0.019 | (-0.046, 0.001) | 0.098 |
| *Indirect effect 12* | -0.012 | (-0.032, 0.004) | 0.210 |
| *Total effect* | -0.189 | (-0.273, -0.113) | <0.001 |
| ***Subgroup II. over 1 year*** |  |  |  |
| *Direct effect* | -0.037 | (-0.095, 0.021) | 0.206 |
| *Indirect effect 1* | -0.001 | (-0.021, 0.019) | 0.905 |
| *Indirect effect 2* | -0.029 | (-0.049, -0.009) | 0.005 |
| *Indirect effect 3* | -0.014 | (-0.035, 0.006) | 0.171 |
| *Indirect effect 4* | 0.004 | (-0.019, 0.027) | 0.740 |
| *Indirect effect 5* | -0.003 | (-0.016, 0.007) | 0.548 |
| *Indirect effect 6* | -0.012 | (-0.028, 0.001) | 0.102 |
| *Indirect effect 7* | -0.041 | (-0.075, -0.011) | 0.012 |
| *Indirect effect 8* | -0.035 | (-0.062, -0.011) | 0.009 |
| *Indirect effect 9* | -0.034 | (-0.062, -0.012) | 0.009 |
| *Indirect effect 10* | -0.008 | (-0.022, 0.003) | 0.197 |
| *Indirect effect 11* | -0.005 | (-0.028, 0.018) | 0.667 |
| *Indirect effect 12* | -0.019 | (-0.040, -0.004) | 0.033 |
| *Total effect* | -0.195 | (-0.259, -0.133) | <0.001 |
| **Resilience → Anxiety** |  |  |  |
| ***Total group*** |  |  |  |
| *Direct effect* | -0.042 | (-0.084, -0.001) | 0.047 |
| *Indirect effect 1* | 0.005 | (-0.007, 0.019) | 0.404 |
| *Indirect effect 2* | -0.008 | (-0.020, 0.003) | 0.180 |
| *Indirect effect 3* | -0.002 | (-0.013, 0.010) | 0.761 |
| *Indirect effect 4* | -0.006 | (-0.021, 0.007) | 0.379 |
| *Indirect effect 5* | -0.002 | (-0.010, 0.006) | 0.658 |
| *Indirect effect 6* | -0.005 | (-0.017, 0.006) | 0.385 |
| *Indirect effect 7* | -0.057 | (-0.080, -0.036) | <0.001 |
| *Indirect effect 8* | -0.012 | (-0.027, 0.001) | 0.072 |
| *Indirect effect 9* | -0.030 | (-0.051, -0.014) | 0.001 |
| *Indirect effect 10* | -0.001 | (-0.008, 0.006) | 0.754 |
| *Indirect effect 11* | -0.008 | (-0.021, 0.004) | 0.189 |
| *Indirect effect 12* | -0.010 | (-0.022, 0.001) | 0.094 |
| *Total effect* | -0.129 | (-0.175, -0.087) | <0.001 |
| ***Subgroup I. 0-1 year*** |  |  |  |
| *Direct effect* | -0.087 | (-0.157, -0.022) | 0.012 |
| *Indirect effect 1* | 0.011 | (-0.008, 0.033) | 0.306 |
| *Indirect effect 2* | -0.008 | (-0.025, 0.007) | 0.332 |
| *Indirect effect 3* | 0.007 | (-0.008, 0.024) | 0.400 |
| *Indirect effect 4* | -0.015 | (-0.038, 0.006) | 0.177 |
| *Indirect effect 5* | -0.001 | (-0.013, 0.013) | 0.937 |
| *Indirect effect 6* | -0.018 | (-0.039, 0.002) | 0.083 |
| *Indirect effect 7* | -0.053 | (-0.087, -0.022) | 0.002 |
| *Indirect effect 8* | -0.001 | (-0.023, 0.020) | 0.892 |
| *Indirect effect 9* | -0.020 | (-0.052, 0.004) | 0.157 |
| *Indirect effect 10* | -0.001 | (-0.013, 0.008) | 0.778 |
| *Indirect effect 11* | -0.008 | (-0.027, 0.010) | 0.402 |
| *Indirect effect 12* | -0.007 | (-0.025, 0.009) | 0.434 |
| *Total effect* | -0.140 | (-0.212, -0.074) | <0.001 |
| ***Subgroup II. over 1 year*** |  |  |  |
| *Direct effect* | -0.001 | (-0.053, 0.048) | 0.960 |
| *Indirect effect 1* | -0.001 | (-0.016, 0.016) | 0.922 |
| *Indirect effect 2* | -0.011 | (-0.028, 0.005) | 0.181 |
| *Indirect effect 3* | -0.010 | (-0.028, 0.007) | 0.260 |
| *Indirect effect 4* | -0.002 | (-0.022, 0.019) | 0.856 |
| *Indirect effect 5* | -0.003 | (-0.014, 0.006) | 0.563 |
| *Indirect effect 6* | 0.005 | (-0.009, 0.020) | 0.480 |
| *Indirect effect 7* | -0.055 | (-0.090, -0.026) | <0.001 |
| *Indirect effect 8* | -0.024 | (-0.043, -0.006) | 0.010 |
| *Indirect effect 9* | -0.040 | (-0.066, -0.020) | <0.001 |
| *Indirect effect 10* | -0.001 | (-0.010, 0.009) | 0.765 |
| *Indirect effect 11* | -0.006 | (-0.023, 0.010) | 0.451 |
| *Indirect effect 12* | -0.010 | (-0.026, 0.003) | 0.172 |
| *Total effect* | -0.120 | (-0.174, -0.067) | <0.001 |
